# Supplementary material for: Direct evaluation of self-quenching behavior of fluorophores at high concentrations using an evanescent field
Source: PLoS One. 2021 Feb 19;16(2):e0247326. doi: 10.1371/journal.pone.0247326 (PMC7895399; doi:10.1371/journal.pone.0247326)
Supplement: S1 File — (DOCX) [file pone.0247326.s001.docx]

**Direct evaluation of self-quenching behavior of fluorophores at high concentrations using an evanescent field**

Wooli Bae*, Tae-Young Yoon and Cherlhyun Jeong*

S1 Note. Calculating the effect of formation of the nonfluorescent dimers at high concentration.

The formation of the dimers decreases the concentration of the free fluorophores.

$\left[ \mathrm{Fluorophore} \right]= \left[ \mathrm{Fluorophore} \right]_{0}-2\left[ \mathrm{Dimer} \right]$ (1)

The concentration of dimers are given by the dissociation constant $K_{D}$

$K_{D}\left[ \mathrm{Dimer} \right]=\left[ \mathrm{Fluorophore} \right]\left[ \mathrm{Fluorophore} \right]$ (2)

By substituting the $\left[ \mathrm{Dimer} \right]$ in the equation (2) using the equation (1), we get

$$\left[ \mathrm{Fluorophore} \right]\left[ \mathrm{Fluorophore} \right]+\frac{K_{D}\left( \left[ \mathrm{Fluorophore} \right]-\left[ \mathrm{Fluorophore} \right]_{0} \right)}{2}=0$$

Solving for the $\left[ \mathrm{Fluorophore} \right]$ gives $\left[ \mathrm{Fluorophore} \right]=\frac{-\frac{K_{D}}{2}\pm\sqrt{\left( \frac{K_{D}}{2} \right)^{2}+2K_{D}\left[ \mathrm{Fluorophore} \right]_{0}}}{2}$

As only a positive concentration is possible,

$$\left[ \mathrm{Fluorophore} \right]=\frac{-\frac{K_{D}}{2}+\sqrt{\left( \frac{K_{D}}{2} \right)^{2}+2K_{D}\left[ \mathrm{Fluorophore} \right]_{0}}}{2}=\sqrt{K_{D}^{2}+8K_{D}\left[ \mathrm{Fluorophore} \right]_{0}}-K_{D}$$

The degree of quenching then becomes

$$\frac{\left[ \mathrm{Fluorophore} \right]_{0}}{[\mathrm{Fluorophore}]}=\frac{\left[ \mathrm{Fluorophore} \right]_{0}}{\sqrt{K_{D}^{2}+8K_{D}\left[ \mathrm{Fluorophore} \right]_{0}}-K_{D}}=\frac{1}{\sqrt{\left( \frac{K_{D}}{\left[ \mathrm{Fluorophore} \right]_{0}} \right)^{2}+8\frac{K_{D}}{\left[ \mathrm{Fluorophore} \right]_{0}}}-\frac{K_{D}}{\left[ \mathrm{Fluorophore} \right]_{0}}}$$

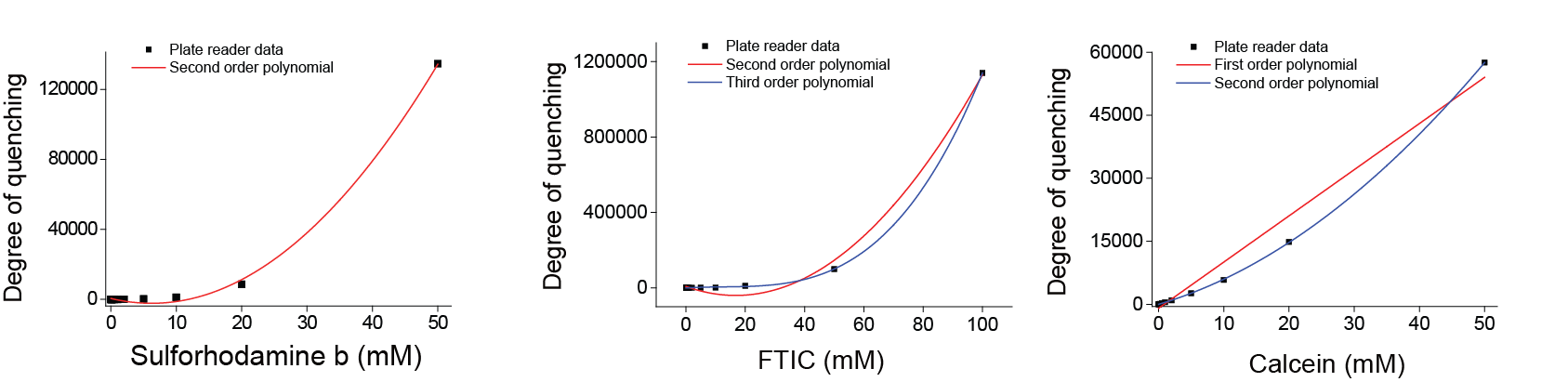


**S1 Figure.** Degree of apparent self-quenching against concentration measured in a plate reader. Data for the FTIC and Calcein could be fitted with higher order polynomial than data from TIRF. Sulforhodamine B was fitted with same second order polynomial.


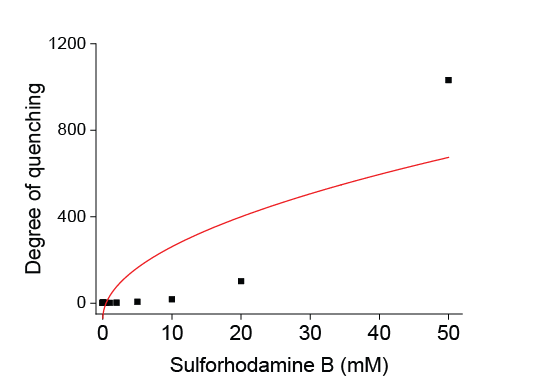


**S2 Figure. Fitting of self-quenching data of sulforhodamine B using equation (2).** The formation of dimer cannot explain the self-quenching behaviour.

**S1 Table.** Raw data for Figure 2.

|  | **Sulforhodamine B** |  |  |
| --- | --- | --- | --- |
| Concentration (mM) | TIR_normalized intensity per dye | Standard error of the mean of TIR_normalized intensity per dye | Plate reader_normalized intensity per dye |
| 100 |  |  |  |
| 50 | 0.00273 | 6.68E-06 | 7.42E-06 |
| 20 | 0.01525 | 2.25E-05 | 1.16E-04 |
| 10 | 0.05547 | 3.31E-05 | 8.50E-04 |
| 5 | 0.16147 | 9.06E-04 | 0.00414 |
| 2 | 0.38807 | 9.02E-04 | 0.02006 |
| 1 | 0.54945 | 0.00106 | 0.05274 |
| 0.5 | 0.75576 | 0.00163 | 0.12611 |
| 0.2 | 0.84551 | 0.00472 | 0.30637 |
| 0.1 | 1 | 0.00791 | 0.45796 |
| 0.05 | 0.94715 | 0.00656 | 0.66242 |
| 0.02 | 0.79927 | 0.02992 | 0.86624 |
| 0.01 | 0.73511 | 0.02036 | 1 |
| 0.005 | 0.64055 | 0.00738 | 0.95541 |
| 0.002 | 0.44526 | 0.01098 | 0.91083 |
| 0.001 | 0.45249 | 0.00291 | 0.91583 |
|  |  |  |  |
|  | **Calcein** |  |  |
| Concentration (mM) | TIR_normalized intensity per dye | Standard error of the mean of TIR_normalized intensity per dye | Plate reader_normalized intensity per dye |
| 100 |  |  |  |
| 50 | 0.0291 | 1.89E-04 | 1.74E-05 |
| 20 | 0.0703 | 5.05E-04 | 6.74E-05 |
| 10 | 0.121 | 0.00166 | 1.72E-04 |
| 5 | 0.199 | 0.00593 | 3.81E-04 |
| 2 | 0.325 | 0.00686 | 0.00105 |
| 1 | 0.45 | 0.00862 | 0.00206 |
| 0.5 | 0.589 | 0.00268 | 0.0041 |
| 0.2 | 0.673 | 0.00353 | 0.01023 |
| 0.1 | 0.685 | 0.03217 | 0.02019 |
| 0.05 | 0.768 | 0.00989 | 0.03938 |
| 0.02 | 0.833 | 0.02064 | 0.08803 |
| 0.01 | 0.865 | 0.01174 | 0.15406 |
| 0.005 | 8.99E-01 | 0.00427 | 0.24011 |
| 0.002 | 1.04E+00 | 0.00302 | 0.40368 |
| 0.001 | 1.00E+00 | 0.00174 | 0.54811 |
| 0.0001 |  |  | 1 |
|  |  |  |  |
|  | **FTIC** |  |  |
| Concentration (mM) | TIR_normalized intensity per dye | Standard error of the mean of TIR_normalized intensity per dye | Plate reader_normalized intensity per dye |
| 100 | 3.63E-04 | 0.0172 | 8.78E-07 |
| 50 | 0.00148 | 0.01539 | 1.00E-05 |
| 20 | 0.00504 | 0.00932 | 9.49E-05 |
| 10 | 0.01171 | 0.0256 | 5.90E-04 |
| 5 | 0.02082 | 0.01318 | 0.00181 |
| 2 | 0.04119 | 0.0064 | 0.00508 |
| 1 | 0.06665 | 0.00981 | 0.01011 |
| 0.5 | 0.10091 | 0.02052 | 0.01961 |
| 0.2 | 0.21535 | 0.0331 | 0.04643 |
| 0.1 | 0.31518 | 0.01095 | 0.08776 |
| 0.05 | 0.42506 | 0.01198 | 0.16707 |
| 0.02 | 0.67412 | 0.0065 | 0.36525 |
| 0.01 | 0.87909 | 0.01199 | 0.57619 |
| 0.005 | 0.94423 | 0.01503 | 0.78598 |
| 0.002 | 1 | 0.02521 | 0.92094 |
| 0.001 | 0.8678 | 0.00141 | 1 |

**S2 Table.** Modified Stern-Volmer equation fit of degree of quenching against concentration.

Sulforhodamine B and FITC were fitted with the following equation.

For $y=c+ax+bx^{\nu} (mM)$,

|  | c | a | b | $\nu$ |
| --- | --- | --- | --- | --- |
| Sulforhodamine B | 1.4 | 0.14 | 0.045 | 2.6 |
| FITC | 2.5 | 8.9 | 0.0016 | 3.0 |
| Calcein | -0.6 | 0.65 | 2.11 | 0.038 |
