# Supplementary material for: Direct evaluation of self-quenching behavior of fluorophores at high concentrations using an evanescent field
Source: PLoS One. 2021 Feb 19;16(2):e0247326. doi: 10.1371/journal.pone.0247326 (PMC7895399; doi:10.1371/journal.pone.0247326)
Supplement: Attachment — Submitted filename: Comments_to_Authors_PONE-D-20-29041.docx [file pone.0247326.s002.docx]

Comments to the authors:

This paper demonstrated a novel method to directly evaluate the self-quenching behavior of soluble fluorophores. Compared to other methods, such as liposome encapsulation or theoretical correction, this method is simpler and direct experimental way to evaluate the self-quenching of fluorophores at arbitrary environment. The actuality of the research topic and the quality of the present study matches the journal’s profile and audience. However, the following comments should be properly addressed before the paper can be considered for publication in *Plos One*.

1. The comparison between the conventional fluorometric system and the nano-cuvette system is not a perfect illustration in Figure 1. The principle and annotation of the TIRF microscopy should be added and discussed.

2. According to what I know, various mechanisms will lead to the self-quenching effect, especially in the high-density fluorophores. In Line 125, the authors mentioned that the plots of sulforhodamine B and FITC exhibited their second-order dependency on concentration. Why do the second-order dependency on concentration indicate the self-quenching occurred via the resonance energy transfer mechanism, but not the collision mechanism between excited fluorophores or the formation of nonfluorescent dimers? Clarify it and add related explanations on the self-quenching reason. Here the authors should spend more time showcasing how the test result determinate the different self-quenching mechanisms.

3. Where is the Conclusion Part for this paper? Add the conclusion for this paper.

4. The depth of the discussion is not strong enough. One would expect some modelling to explain the different self-quenching mechanisms.
